# Evaluating the Readability and Quality of Bladder Cancer Information from AI Chatbots: A Comparative Study Between ChatGPT, Google Gemini, Grok, Claude and DeepSeek

**DOI:** 10.3390/jcm14217804

**Published:** 2025-11-03

**Authors:** Kunjan Patel, Robert Radcliffe

**Affiliations:** Royal Derby Hospital, Derby DE22 3NE, UK; robert.radcliffe@nhs.net

**Keywords:** bladder cancer, AI chatbot, readability matrix, LLM

## Abstract

**Background/Objectives**: Artificial Intelligence (AI)-based chatbots such as ChatGPT are easily available and are quickly becoming a source of information for patients as opposed to traditional Google searches. We assessed the quality of information on bladder cancer, provided by various AI chatbots such as ChatGPT 4o, Google Gemini 2.0 flash, Grok 3, Claude Sonnet 3.7 and DeepSeek R1. Their responses were analysed in terms of Readability Indices, and two consultant urologists rated the quality of information provided using the validated DISCERN tool. **Methods**: The top 10 most frequently asked questions about bladder cancer were identified using Google Trends. These questions were then provided to five different AI chatbots, and their responses were collected. No prompts were used, reflecting natural language queries that patients would use. The responses were analysed in terms of their readability using five validated indices: Flesch Reading Ease (FRE), the Flesch–Kincaid Reading Grade Level (FKRGL), the Gunning Fog Index, the Coleman–Liau Index and the SMOG index. Two consultant urologists then independently assessed the responses of various AI chatbots using the DISCERN tool, which rates the quality of the health information on a five-point LIKERT scale. Inter-rater agreement was calculated using Cohen’s Kappa and the intraclass correlation coefficient (ICC). **Results**: ChatGPT 4o was the overall winner in readability scores, with the highest Flesch Reading Ease score (59.4) and the lowest average reading grade level (7.0) required to understand the material. Grok 3 was a close second (FRE 58.3, grade level 8.7). Claude 3.7 Sonnet used the most complex language in its answers and therefore scored the lowest FRE score of 44.9, with the highest grade level (9.5) and also the highest complexity on other indices. In the DISCERN analysis, Grok 3 received the highest average score (52.0), followed closely by ChatGPT 4o (50.5). The inter-rater agreement was highest for ChatGPT 4o (ICC: 0.791; Kappa: 0.437), while it was lowest for Grok 3 (ICC: 0.339, Kappa 0.0, Weighted Kappa 0.335). **Conclusions**: All AI chatbots can provide generally good-quality answers to questions about bladder cancer with zero hallucinations. ChatGPT 4o was the overall winner, with the best readability metrics, strong DISCERN ratings and highest inter-rater agreement.

## 1. Introduction

The internet is becoming the primary source of health information for many patients, and bladder cancer is no exception. Bladder cancer is characterised by high recurrence rates, especially in non-muscle invasive disease, and the need for often lifelong surveillance [1,2]. This chronic relapsing course often generates significant anxiety and psychological distress [3] and repeated information-seeking behaviour among patients. A global survey of patient and carer experiences with bladder cancer suggested that almost 82% respondents needed more information at the time of their diagnosis [4]. Therefore, it is not surprising that patients are using the internet to find information about bladder cancer. There are studies looking at how the general public uses online search tools such as Google to access health-related information [5,6,7]. With their widespread availability, beginning with ChatGPT in 2022, the use of Artificial Intelligence chatbots is rapidly replacing traditional Google searches. In fact, Google’s share of global market searches has declined to below 90% for the first time since 2015 with the growth of AI chatbots. Medical researchers are therefore beginning to assess the efficacy of AI chatbots in responding to health-related inquiries including in Urology [8]. However, a key problem with using AI chatbots for medical information is their tendency to hallucinate or generate false answers [9,10,11]. Previous research has mainly focused on ChatGPT for medical information delivery [12,13,14]. Yet, to our knowledge, no study has yet assessed the quality of bladder cancer information generated from various AI chatbots nor compared its readability and accuracy from the perspective of a urologist.

## 2. Materials and Methods

### 2.1. Question Selection and Prompting Process

Google Trends was used to define the 10 most commonly asked questions about bladder cancer by the public. A “worldwide” filter was used with a 12-month lookback period. Google Trends provides aggregated, de-identified search frequencies but does not supply demographic information. These questions therefore represent high-volume global public concerns but cannot be assumed to be fully representative of all demographic groups. The questions were then inputted in ChatGPT 4o), Google Gemini 2.0 Flash), Grok 3 Claude Sonnet 3.7 and DeepSeek R1. We submitted each question once to each model using default settings to ensure direct comparability. We did not conduct repeated runs or adjust “temperature/creativity” parameters, which means that output results capture a single output instance per model. Zero-shot prompting was used, where each of the ten questions was input verbatim into the models without providing sample answers, so that the responses reflected the model’s baseline capability to answer natural patient queries. We recognise that prompt engineering, clarifying follow up questions or chain-of-thought instructions can markedly influence output readability and accuracy. We intentionally used the *exact* top 10 bladder cancer questions identified on Google Trends to ensure that the inputs reflected the most common public queries at the time of study. Although this approach standardises comparison across models, it does not capture the full range of ways patients may phrase similar questions (e.g., synonyms, colloquialisms or partial phrases). We therefore interpret our findings as a baseline analysis of common queries rather than an exhaustive test of linguistic variability.

### 2.2. Readability Assessment

The various AI chatbot outputs were individually inputted into the Readability Formulas website (https://readabilityformulas.com, accessed on 16 August 2025), which is a free open-source tool. We reported the Flesch–Kincaid Reading Ease (FRE), Flesch–Kincaid Reading Grade Level (FKRGL), Gunning Fog Index, Coleman–Liau Index and SMOG index, which are validated tools for readability assessment. For FRE scores, higher is better, while for FKRGL, Gunning Fog, Coleman–Liau and SMOG, a lower value corresponds to an easier text to read.

### 2.3. Urologist Assessment of AI Chatbot Generated Answers

Two consultant urologists then independently assessed the quality of various AI chatbot answers using DISCERN tool (DISCERN) to judge the reliability of answers as a source of information about treatment choices and about where the sources of said evidence are explicit. Inter-rater agreement was measured using validated tools like Cohen’s Kappa and intraclass correlation coefficient (ICC).

## 3. Results

**Figure 1:**
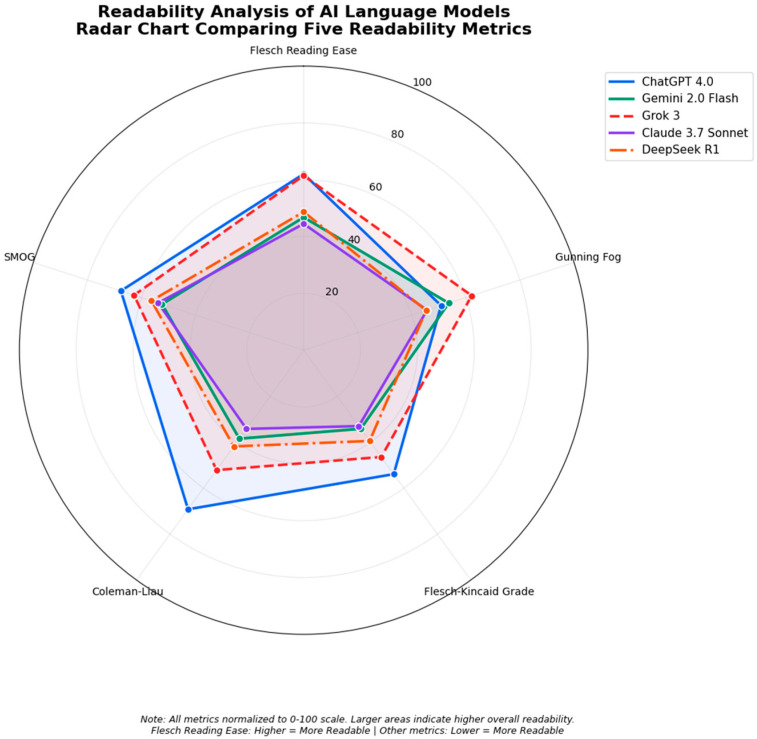
Readability analysis of AI language model radar chart.

A summary of the readability assessments for different AI chatbots is provided in Table 1 and Figure 1. ChatGPT 4o was the winner in overall readability scores for all questions, with an average FRE score of 59.4 and the lowest average grade level of 7 needed to understand the material provided. Grok 3 was a close runner up, with an average FRE score of 58.3 and a grade level of 8.7. Claude 3.7 Sonnet was last in readability scores due to the complex medical language used in its answers and had the lowest average FRE score (44.5) among its answers and the highest complexity scores among other indices. Google Gemini 2.0 Flash and DeepSeek R1 had scores in between these, with an average FRE of 47.9 and an FKRGL of 9.375 for Gemini 2.0 and an average FRE of 47.5 and an average FKRGL of 9.149 for Deepseek R1.

### Urologist Assessment of AI Chatbot Answers

- Two consultant urologists independently assessed the accuracy, completeness and clarity of the answers for each of the 10 questions for bladder cancer using the DISCERN tool, which is a clinically validated scoring system for assessing the quality of written information on treatment choices for a health problem devised by the University of Oxford [15]. Table 2 shows the summary of their average DISCERN scores. As demonstrated, Grok 3 received the highest average DISCERN score of 52, followed closely by ChatGPT at 50.5. Gemini Flash 2.0 had the lowest average DISCERN score for the quality of answers at 47.
- Both urologist reviewers independently recorded whether each response contained any factual inaccuracies. No hallucinations were detected across 50 chatbot answers (0/50; κ = 1.0).

**Table 2:** Average DISCERN scores for overall quality of information by two consultant urologists.

Question	ChatGPT 4.0		Gemini Flash 2.0		Grok 3		Claude Sonnet 3.7		Deepseek R1	
Reviewer	1	2	1	2	1	2	1	2	1	2
1	4	4	4	4	4	4	4	3	4	4
2	5	3	5	4	5	4	4	3	5	4
3	4	4	3	4	3	4	3	4	3	5
4	1	1	1	1	1	2	1	1	1	1
5	1	1	1	1	1	4	1	1	1	1
6	4	5	4	4	3	4	4	4	3	4
7	1	1	1	1	1	3	2	1	1	2
8	2	2	2	3	2	3	3	2	3	2
9	5	5	5	3	4	4	4	3	5	3
10	5	4	4	4	4	4	5	3	5	4
11	4	3	4	2	3	4	5	3	4	4
12	3	4	3	2	2	4	4	2	3	1
13	4	2	3	2	3	2	4	3	2	2
14	4	4	3	5	4	4	4	3	3	4
15	3	2	3	2	3	4	3	3	3	3
16	3	3	3	3	3	4	4	3	3	3
Total	**53**	**48**	**49**	**45**	**46**	**58**	**55**	**42**	**49**	**47**

The analysis of the agreement was calculated using Cohen’s Kappa, the Weighted Kappa and the interclass correlation coefficient (ICC) using Python 3.13. A summary table of the agreement statistics is shown in Table 3.

## 4. Discussion

As far as the authors are aware, this is the first time that various large language model outputs have been compared to ChatGPT in terms of their output as a provider of information for patients diagnosed with bladder cancer. The results indicate that while ChatGPT remains the overall winner in terms of readability and the quality of medical information provided, all the other chatbots are not far behind and provide almost similar levels of coherent answers, which are easily comprehensible. A notable exception was Claude 3.7, which is generally used for complex tasks related to writing software code and may not be trained for the tasks that we assigned to it. Most chatbots were also aware of their own limitations in terms of the answers provided and frequently reiterated the need to contact a healthcare provider with any further questions. The only chatbot to reliably reveal sources of information without needing to prompt it was Google Gemini 2.0 flash, while every other chatbot did provide sources on request. Also, Gemini was the only chatbot that was aware of our geographical location (in the UK) and provided mainly NHS and NICE sources, while other chatbots all used sources from the USA for their information. This probably reflects the locations of these software companies, except DeepSeek which is based in China. This regional bias could lead to a guideline mismatch if recommendations differ by country. This may become more relevant in the future as the treatments suggested by AI chatbots may not necessarily be available in each country and may not even be approved for the suggested indication in the patient’s own country. Developers should therefore consider geo-sensitive citation filters or clearly display the geographic location of each cited source so users can verify the local relevance. Furthermore, there were no images provided, even as explanations for complex medical procedures like a radical cystectomy or TURBT, to further supplement and reinforce the information that was dispensed in these responses. This continues to be an ongoing important limitation in the data provided by chatbots. While all chatbots have various image generation tools, none can currently combine text answers with relevant images in the answer unless a specific image is asked for by the user.

Generative AI remains a contentious but exciting area for the medical community, given how far reaching the implications are for the profession as a whole [16]. Indeed, a search for “LLM” and “Urology” reveals almost 2120 publications since 2021 (when ChatGPT was first released to the public). Multiple publications have shown LLMs’ ability to pass Medical Licencing Exams and speciality board exams including Urology [17,18,19,20,21,22]. Zhu et al. [23] demonstrated the ability of various AI chatbots to provide answers to medical questions about prostate cancer. The reported accuracy and comprehensiveness of ChatGPT for prostate cancer was greater than 90%. Our responses indicate that almost all AI chatbots are accurate when it comes to the quality of medical information provided, and the only differences arise mainly due to semantics and the lack of depth in the answers provided. It could be argued that an AI chatbot trained exclusively on medical data and papers such as MedPaLM (https://sites.research.google/med-palm/, accessed on 16 August 2025) may be more suited to medical questions, although this chatbot is currently not available for public use.

Ultimately, despite demonstrations that AI LLMs can gain sufficient knowledge to pass medical exams, the primary purpose for using LLMs as a urologist would be to help in administrative tasks and in answering patient queries, thus reducing administrative burdens for clinicians and allowing them to focus on more “human” elements of their role. It remains to be seen whether patients would accept answers provided by a chatbot rather than their own clinician. An AI chatbot can also not replace the psychological competency and empathy of a human clinician.

Our findings highlight that readability scores alone cannot determine the suitability of AI-generated health information. A text that is highly readable but factually incorrect poses a greater risk than a more complex but accurate answer. In clinical communication, accuracy must take precedence; once correctness is assured, efforts should focus on simplifying language to a reading level accessible to the general public (ideally around 6th–8th-grade in English). Clinicians should therefore view readability metrics as a secondary optimisation target and counsel patients that an easily understood answer is not necessarily a correct one.

Our study has several limitations. Firstly, the responses generated by AI chatbots can vary greatly depending on how the questions are formulated. Large language models are known to exhibit prompt sensitivity, and small lexical changes can alter readability and quality. Future work could employ a thesaurus-based or semantic–paraphrase framework to test multiple variants of each question and quantify the output variability. Also, large language models are stochastic; repeat queries can yield different answers. An important limitation is the absence of repeated runs or prompt variations, which would allow for the estimation of the output variability. Our findings should, therefore, be interpreted as a baseline snapshot of each model’s typical performance. Therefore, any future studies should perform multiple independent runs and incorporate a prompt–paraphrase sensitivity analysis to characterise model stability and the effect of the input phrasing. Secondly, specialist prompts often contain precise medical terminology, whereas public queries are typically shorter, symptom-based or colloquial. Large language models may generate different levels of detail, readability and source citation depending on this input style.

Our analysis focused on high-frequency public questions; future work should compare outputs generated from specialist-level prompts and layperson prompts for the same clinical scenarios to better characterise this variability. Thirdly, although the responses were assessed by experienced urologists using DISCERN, the responses are still subjective and there are no “gold-standard” answers to any questions. While DISCERN captures the reliability and quality of the treatment information, it does not directly measure source transparency (JAMA benchmark tool), overall web-page quality (Global Quality Score) or third-party accreditation (HON). Future studies could incorporate these complementary tools to provide a more comprehensive assessment Fourthly, only two consultant urologists independently assessed the chatbot outputs. While both have >10 years of clinical experience in bladder cancer management, we recognise that a larger and more diverse reviewer panel (including nurses, patients and other urologists) would further enhance the external validity. Our study should therefore be viewed as an exploratory pilot analysis rather than a definitive consensus evaluation. Finally, all LLMs used in this study tend to have a knowledge cut-off date, which might limit their ability to answer with up-to-the-moment data.

The authors further acknowledge that ten high-frequency questions cannot capture the full spectrum of patient concerns across different age, gender and cultural backgrounds. Future studies should identify and incorporate a larger, demographically stratified question bank drawn from patient forums, clinic records and qualitative interviews.

Our analysis focused on the textual characteristics of the chatbot outputs but did not measure patient-reported outcome measures, such as comprehension, trust or decision making. These human factors are critical for determining the clinical utility and safety of AI-generated information. Future work should incorporate prospective patient studies, for example, by exposing patients to chatbot responses and analysing their understanding, credibility, decision conflict and intended actions.

## 5. Conclusions

This study provides insight into using a variety of LLMs apart from ChatGPT to answer questions about bladder cancer diagnosis. This study shows that overall, all current commonly used AI chatbots are capable of answering patient queries about bladder cancer with accurate and consistent answers with an awareness of their own limitations and exhortations to contact their primary (human!) healthcare provider.

## Figures and Tables

**Table 1:** Readability assessments scores.

Q#	ChatGPT 4.0					Gemini 2.0 Flash					Grok 3					Claude 3.7 Sonnet					DeepSeek R1				
	FRE	GF	FK	CL	SM	FRE	GF	FK	CL	SM	FRE	GF	FK	CL	SM	FRE	GF	FK	CL	SM	FRE	GF	FK	CL	SM
1	45	11.9	9.43	12.9	8.76	38	11.6	10.05	14.48	8.17	28	14.4	14.63	15.84	12.69	32	14	13.6	15.5	11.9	32	14.1	12.1	15.5	10.6
2	49	9.9	8.91	11.2	7.7	51	10.1	8.89	10.79	8.09	51	9.9	11.25	11.28	9.62	58	9.3	7.62	10.5	7.3	53	9.9	7.96	11.6	7.09
3	65	9.1	6.55	8.05	7.24	50	10.6	8.45	11.03	7.78	67	8.3	8.19	8.83	7.9	49	11	9.05	12.3	8.47	54	10.6	8.37	10.7	8.25
4	68	9.8	5.59	7.52	6.81	57	9.4	7.5	9.78	7.58	64	8.8	8.32	10.26	7.88	52	11	8.79	11.8	8.83	55	11	8.1	10.4	8.4
5	56	11.5	7.7	10.1	8.17	42	11.3	9.96	13.04	8.71	59	9.2	7.68	11.38	7.5	26	16	12	15.9	10.4	25	14.5	11.7	15.1	9.23
6	58	11.8	7.53	9.49	8.26	55	9	8.91	11.1	7.91	65	9.6	7.47	9.81	7.99	50	12	9.03	11.9	8.95	60	9.6	7.01	9.9	7.15
7	52	12.5	8.14	10.2	8.51	46	11.7	10.05	11.47	9.81	62	9.9	7.98	10.66	8.52	47	11	9.06	11.1	8.68	45	13.1	9.64	11.8	9.54
8	65	10.7	6.41	9.19	7.76	43	10.4	11.44	12.89	9.47	63	9.6	7.2	10.19	7.58	46	10	9.13	13.5	7.82	53	10.4	8.37	11.2	7.89
9	66	10.5	6.34	7.89	7.63	46	10.4	9.54	12.3	8.19	61	9.7	7.8	10.78	7.9	37	12	10.1	13	8.22	42	12.8	9.95	12.3	9.06
10	68	10.1	6.38	8.66	7.94	51	10.2	8.96	12.19	8.4	63	9.8	8.31	11.27	8.56	48	12	11.9	12.5	10.1	56	9.5	8.29	11.8	8.09

**Table 3:** Agreement scores between reviewers for various AI chatbots.

Model	ICC2 (Agreement)	Cohen’s Kappa	Weighted Kappa (Quadratic)
ChatGPT 4.0	0.791	0.437	0.780
Gemini Flash 2.0	0.657	0.291	0.643
Grok 3	0.339	0.000	0.325
Claude Sonnet 3.7	0.562	0.063	0.546
Deepseek R1	0.669	0.308	0.655

## Data Availability

All data used in this analysis are available in the Supplementary File.

## Supplementary Materials

The following supporting information can be downloaded at https://www.mdpi.com/article/10.3390/jcm14217804/s1; Table S1: link to the AI chatbots used in the study; Table S2: Questions asked to the AI chatbots

## Abbreviations

The following abbreviations are used in this manuscript:

LLM	Large Language Model
FRE	Flesch Reading Ease
AI	Artificial Intelligence
FKRGL	Flesch–Kincaid Reading Grade Level
