# Supplementary material for: Evaluating the Readability and Quality of Bladder Cancer Information from AI Chatbots: A Comparative Study Between ChatGPT, Google Gemini, Grok, Claude and DeepSeek
Source: J Clin Med. 2025 Nov 3;14(21):7804. doi: 10.3390/jcm14217804 (PMC12610445; doi:10.3390/jcm14217804)
Supplement: Supplementary file 1 [file jcm-14-07804-s001.zip › jcm-3851990-supplementary.pdf]

Table S1. Link to the AI chatbots used in the study

Model	Version/Release	URL/Interface
ChatGPT-4o	May 2025 release	https://chat.openai.com
Google Gemini 2.0 Flash	June 2025	https://gemini.google.com
Grok 3	June 2025	https://grok.com
Claude Sonnet 3.7	June 2025	https://claude.ai
DeepSeek R1	June 2025	https://deepseek.com

Table S2. Questions asked to the AI chatbots.

	Question
1	what are the causes of bladder cancer?
2	What are symptoms of bladder cancer ?
3	I have blood in urine. What should I do?
4	What are the tests for bladder cancer?
5	What are the treatments for bladder cancer?
6	What is a TURBT?
7	What is a cystectomy ?
8	What is BCG in bladder cancer?
9	what is mitomycin-C in bladder cancer?
10	can you die from bladder cancer?
